# Linoleic acid as corpse recognition signal in a social aphid

**DOI:** 10.1186/s40851-021-00184-w

**Published:** 2022-01-06

**Authors:** Harunobu Shibao, Mayako Kutsukake, Shigeru Matsuyama, Takema Fukatsu

**Affiliations:** 1 Graduate School of Life and Environmental Sciences, University of Tsukuba, Tsukuba, 305-8572 Japan; 2 Bioproduction Research Institute, National Institute of Advanced Industrial Science and Technology (AIST), Tsukuba, 305-8566 Japan; 3 Department of Biological Sciences, Graduate School of Science, The University of Tokyo, Tokyo, 113-0033 Japan

**Keywords:** Social aphid, Soldier caste, *Tuberaphis styraci*, Corpse recognition signal, Death pheromone, Fatty acid, Linoleic acid, Cleaning behavior, Insect gall

## Abstract

Social insect colonies constantly produce dead insects, which cause sanitary problems and potentially foster deadly pathogens and parasites. Hence, many social insects have evolved a variety of hygienic behaviors to remove cadavers from the colonies. To that end, they have to discriminate dead insects from live ones, where chemical cues should play important roles. In ants, bees and termites, such corpse recognition signals, also referred to as “death pheromones” or “necromones”, have been identified as fatty acids, specifically oleic acid and/or linoleic acid. Meanwhile, there has been no such report on social aphids. Here we attempted to identify the “death pheromone” of a gall-forming social aphid with second instar soldiers, *Tuberaphis styraci*, by making use of an artificial diet rearing system developed for this species. On the artificial diet plates, soldiers exhibited the typical cleaning behavior, pushing colony wastes with their heads continuously, against dead aphids but not against live aphids. GC-MS and GC-FID analyses revealed a remarkable increase of linoleic acid on the body surface of the dead aphids in comparison with the live aphids. When glass beads coated with either linoleic acid or body surface extract of the dead aphids were placed on the artificial diet plates, soldiers exhibited the cleaning behavior against the glass beads. A series of behavioral assays showed that (i) soldiers exhibit the cleaning behavior more frequently than non-soldiers, (ii) young soldiers perform the cleaning behavior more frequently than old soldiers, and (iii) the higher the concentration of linoleic acid is, the more active cleaning behavior is induced. Analysis of the lipids extracted from the aphids revealed that linoleic acid is mainly derived from phospholipids that constitute the cell membranes. In conclusion, we identified linoleic acid as the corpse recognition factor of the social aphid *T. styraci*. The commonality of the death pheromones across the divergent social insect groups (Hymenoptera, Blattodea and Hemiptera) highlights that these unsaturated fatty acids are generally produced by enzymatic autolysis of cell membranes after death and therefore amenable to utilization as a reliable signal of dead insects.

**Supplementary Information:**

The online version contains supplementary material available at 10.1186/s40851-021-00184-w.

## Introduction

In social insect colonies, a large number of individuals are living together within a limited nest space at a high population density, where dead insects are constantly produced. Accumulation of the insect corpses is not only obstructive but also dangerous in that the cadavers may potentially host or even foster deadly pathogens and parasites, and thus must be promptly removed from the colonies. Hence, the social insects have evolved a variety of hygienic behaviors, so-called necrophoresis or undertaking behavior, which comprise an important element of social immunity [1–3].

Eusocial insects like bees, ants and termites are characterized by the presence of reproductive, morphological and behavioral division of labor with specialized caste individuals called reproductives, workers, soldiers, etc. [4–6], among which the undertaking behavior is usually performed by worker individuals [3]. Although relatively less known, some aphids are also social with specialized caste individuals called reproductives and soldiers [7–9]. These aphids parthenogenetically produce soldier nymphs that perform colony defense against natural enemies. Notably, soldiers of gall-forming aphids often perform additional social tasks such as gall cleaning [10–12], gatekeeping [13, 14] and gall repair [15–18].

*Tuberaphis styraci* is a social aphid that forms a large coral-shaped gall, up to 12 cm in diameter and hosting over 20,000 insects in maturity, on the tree *Styrax obassia*. In the gall, monomorphic first instar nymphs differentiate into reproductive nymphs and soldier nymphs upon second instar molt. While reproductive nymphs grow to adult and reproduce, soldiers neither grow nor reproduce but perform social tasks, namely colony defense against natural enemies and nest cleaning by disposing colony wastes [12, 19]. Upon encountering other insects, soldiers attack them by stinging with the stylet, whereas soldiers push wax-coated honeydew globules, shed skins and aphid cadavers with their head out of the exit openings on the underside of the gall (Movies 1 and 2). *T. styraci* provides a unique model for studies on the aphid sociality owing to the establishment of an artificial diet rearing system [20], by which the mechanisms underlying the density-dependent caste differentiation and regulation have been experimentally investigated in detail [21–26].

For performing undertaking behavior properly, social insect workers discriminate dead colony mates from live ones based on behavioral, visual, and chemical cues, where chemical factors often play important roles. In some cases, such corpse recognition factors, also referred to as “necromones” or “death pheromones”, are identified as fatty acids, specifically oleic acid and/or linoleic acid, in ants [27–30], bees [31, 32] and termites [33, 34]. Thus far, no such corpse recognition factors have been identified in social aphids. In this study, therefore, we attempted to identify hitherto unknown “death pheromone” in an aphid social system by making use of the artificial diet rearing system of *T. styraci*.

## Materials and methods

### Insect collection, sampling, and rearing on artificial diet

A total of ten galls (#1–10) of *T. styraci* were collected from the host tree *S. obassia* at Shomaru, Saitama, Japan, or Minakami, Gumma, Japan, and brought to the laboratory. The insects were collected from the galls, sorted under a dissection microscope, and maintained on the artificial diet rearing system as described previously [20, 26].

### Insect sampling, sorting and staging

The collected insects were sampled, sorted and staged as described [26].

Nymphs and adults. Normal first, second, third and fourth instar nymphs, second instar soldiers, and adults were identified based on their size and morphology.

Non-soldier nymphs. Normal second to fourth instar nymphs (excluding first instar nymphs) were defined as non-soldier nymphs.

Young adults and old adults. For obtaining age-defined adult populations, many fourth instar nymphs were kept on artificial diet plates and inspected every day, and newly-molted adults were collected and transferred to new diet plates. Adult insects within 7 days after molt were defined as young adults, whereas adult insects no less than 8 days after molt were defined as old adults.

New soldiers, young soldiers and old soldiers. For obtaining age-defined soldier populations, many first instar nymphs were kept on artificial diet plates and inspected every day, and newly-molted soldiers were collected and transferred to new diet plates. Soldiers within one day after molt, 10 days after molt and 20 days after second instar molt were defined as new soldiers, young soldiers and old soldiers, respectively.

### Observation of cleaning behavior against dead aphids

Young soldiers were individually placed on an artificial diet arena and presented with 30 live old adults or 30 dead old adults that had been kept for 0, 1, 3 and 7 days at room temperature. The dead aphids were prepared by killing live aphids in a freezer at − 20 °C. For assaying the cleaning behavior, each insect on an artificial diet arena was continuously observed for 10 min under a dissection microscope. When an insect continuously pushed a test object (live colony mate or colony mate corpse) for 5 s or longer, the insect was regarded as performing cleaning behavior. For each of five test arenas, a total of 30 insects, which consisted of 10 young soldiers from 3 different galls (#1–3), were subjected to the behavioral observations.

### Analysis of fatty acids

Cuticular fatty acids were extracted from live aphids (groups of ten old adults or thirty old soldiers) and dead aphids (groups of ten old adults or thirty old soldiers that were 0, 1, 3 and 7 days after freeze-killing) by soaking for 1 min in 25 μl hexane containing 10 ng/μl erucic acid (absent in the insect extracts) as an internal standard. The fatty acids, including the internal standard, in the crude extracts were converted to methyl esters via reaction with diazomethane for identification by gas chromatography-mass spectrometry (GC-MS) and quantitative determination by gas chromatography with a flame ionization detector (GC-FID). Each of the samples was dried under a nitrogen stream and re-suspended in 5 μl of hexane, and 1 μl aliquots of the solvent were subjected to the analyses. The identification of fatty acids was conducted by comparing retention times and mass spectra with those of authentic reference compounds. For quantification of fatty acids in each sample, individual peak areas were compared to the peak area of the internal standard. For comparing the fatty acid profiles, the relative proportion of each compound to the total fatty acids (excluding the internal standard) was calculated for each sample. The extraction and quantification of the cuticular fatty acids of the live aphids and dead aphids of each caste with different post-mortem times were conducted with 6 replicates (= 2 replicates × 3 galls [#1–3]).

### Analysis of lipids

After the hexane extraction of their cuticular fatty acids, the aphids were homogenized, and their body lipids were extracted two times with 500 μl of chloroform/methanol (2:1 v/v) for a day. The extracts were combined, dried, and chromatographed on a 500 mg silica gel column (Wako-gel C-300, Wako, Tokyo, Japan) with stepwise elution with 5 ml each of ether (two times) and methanol (two times), by which neutral lipids and phospholipids were recovered in the ether and methanol fractions, respectively. To determine the fatty acid compositions of the two lipid classes, each of the fractions was dried, re-suspended in 25 μl of hexane containing an internal standard (10 ng/μl erucic acid), and divided into two equal parts. One of them was reacted with diazomethane to evaluate the amounts of free fatty acids, and the other one was subjected to an alkaline hydrolysis with 5% KOH/ethanol followed by diazomethane reaction to evaluate the neutral lipid fatty acid composition or phospholipid fatty acid composition. The extraction, fractionation, and reaction to evaluate the neutral lipid/phospholipid fatty acid composition of live aphids were conducted with 2 replicates (= 1 replicate × 2 galls [#4–5]). Each of the samples was dried and re-suspended in 5 μl of hexane, and 1 μl aliquots were subjected to GC-MS and GC-FID analyses, respectively, as described below.

### GC-FID

GC-FID was conducted on an HP6890 (Hewlett-Packard, Palo Alto, CA, U.S.A.) equipped with a bonded-phase FFAP column (25 m × 0.25 mm, 0.25 μm film thickness). Samples were injected in the splitless mode (sampling time: 1 min) at an injection port temperature of 280 °C. Helium was used as a carrier gas at a flow rate of 1 ml/min in the constant flow mode. The oven temperature was set at 70 °C for 1 min, then raised to 280 °C at a rate of 8 °C/min, and held at the final temperature for 10 min. A flame-ionization detector (FID) was operated at 280 °C and the chromatograms were analyzed by an HP ChemStation software.

### GC-MS

Mass spectra were obtained by GC-MS. Samples were injected into an HP6890N gas chromatograph operated under the same condition for GC-FID, except that the column (a fused silica HP-1MS column, 30 m × 0.25 mm, 0.25 μm film thickness) outlet was introduced at 280 °C into an MS-600H mass spectrometer (JEOL, Tokyo, Japan). Temperature of the ionization chamber was set at 190 °C and the ionization was done in the electron impact mode at 70 eV. The data were acquired in the scan mode (scan range 40–600 amu; scan speed 0.29 s) and analyzed by a TSS2000 software (ver. 2.00, Shrader Analytical and Consulting Laboratories Inc., Detroit, MI, USA).

### Behavioral assay against glass beads treated with fatty acids and aphid extracts

The insects were individually placed on an artificial diet arena with 300 glass beads (0.4 mm in diameter) that had been soaked in 20 μl of crude hexane extract or 20 μl of hexane solution containing test compound(s) and then air-dried, and their behaviors against the glass beads were observed (Fig. 5a). For obtaining crude hexane extract, about 500 old adults (3 days or longer after freeze-killing) were extracted with 1 ml of hexane for 1 min, and the extract solution was concentrated to the final volume of 20 μl. The six most abundant compounds, lauric acid (C12:0), myristic acid (C14:0), palmitic acid (C16:0), stearic acid (C18:0), oleic acid (C18:1) and linoleic acid (18:2), were tested alone (3000 ng each) or blended at the ratios of 0.6: 0.8: 1.1: 0.8: 1.0: 1.0 (= 1800 ng lauric acid + 2400 ng myristic acid + 3300 ng palmitic acid + 2400 ng stearic acid + 3000 ng oleic acid + 3000 ng linoleic acid), mimicking the cuticular fatty acid profile of 1-week-old dead adult aphids. The final concentrations of the test samples applied to individual glass beads were adjusted to 10 ng or 1 ng linoleic acid per bead with or without other compounds. Control glass beads were treated with pure hexane. For assaying cleaning behavior, each insect on an artificial diet arena was observed for 10 min under a dissection microscope. When an insect continuously pushed a glass bead for 5 s or longer, the insect was regarded as performing cleaning behavior. For each assay, a total of 50 insects, which consisted of 15–20 aphids in each of the six caste/age classes (non-soldier nymphs, young adults, old adults, new soldiers, young soldiers, and old soldiers) from 3 different galls (#6–8), were subjected to the behavioral observations.

### Behavioral assay against live and dead aphids treated with linoleic acid

Live and freshly killed non-soldier aphids, which consisted of third instar nymphs, fourth instar nymphs and adults, were randomly divided into groups and used for the experiments. The following seven treatment groups were prepared: intact live insects as negative control (10 intact non-soldier aphids plus 100 young soldiers per arena); live insects treated with hexane as solvent control (10 hexane-treated non-soldier aphids plus 100 young soldiers per arena); live insects treated with linoleic acid (10 non-soldier aphids treated with linoleic acid plus 100 young soldiers per arena); freshly killed insects as negative control (10 dead non-soldier aphids plus 100 young soldiers per arena); freshly killed insects treated with hexane as solvent control (10 hexane-treated dead non-soldier aphids plus 100 young soldiers per arena); freshly killed insects treated with linoleic acid (10 dead non-soldier aphids treated with linoleic acid plus 100 young soldiers per arena); and dead insects 3 days after killing as positive control (10 dead non-soldier aphids plus 100 young soldiers per arena). Application of 0.2 μl hexane containing 10 ng linoleic acid or 0.2 μl pure hexane was conducted onto the dorsal surface of each insect using a 5 μl micro-syringe. For behavioral assay, 10 non-soldier test insects were introduced into an artificial diet arena containing 100 young soldiers, and observed under a dissection microscope for 30 min continuously. When a non-soldier insect was continuously pushed by soldier(s) for 5 s or longer, the insect was regarded as “cleaned”. Each assay was replicated 4 times (= 2 times × 2 galls [#9–10]), and the data were pooled to calculate the proportion of the test insects cleaned by soldier(s) per total number of the test insects.

### Microwave heat treatment

In order to suppress autolytic production of fatty acids in aphid cadavers, groups of ten old adults were killed in a freezer, then heated in a microwave oven for 1 min at 600 W, and kept at room temperature for 0, 1, 2 and 3 days. Control groups were not subjected to heat treatment after freeze-killing. Cuticular fatty acid profiles of the two experimental groups were compared in a time course. Each treatment was replicated 6 times (= 2 times × 3 galls [#1–3]).

### Statistics

Statistical analyses were performed using R software ver. 4.0.4. For the behavioral assessment, the proportions of the soldiers that exhibited cleaning behavior against live colony mates or colony mate corpses were compared among treatments, using generalized linear mixed models (GLMM) with sequential Bonferroni (*P* < 0.05). For each model, the proportion of cleaning soldiers was regarded as the response variable, assuming a binomial distribution (logistic transformation). Treatments were used as an explanatory variable and colonies were incorporated into the model as a random effect. To test the differences in linoleic acid quantity between the live and dead adults and between the live and dead soldiers, we used Wilcoxon rank-sum tests. When testing differences in the linoleic acid quantity between the dead adults with or without microwave heating treatment over time, we performed Steel’s multiple comparison test or Wilcoxon rank-sum tests for each time point separately. For the bioassay experiment testing for each caste’s dose-dependency and cleaning activity, the proportions of the aphids that exhibited cleaning behavior against glass beads coated with linoleic acid or aphid surface extract were compared with those of the counterpart control aphids that were presented with solvent-coated glass beads using GLMM with sequential Bonferroni (*P* < 0.05). The proportion of the cleaning aphids was regarded as the response variable, assuming a binomial distribution. Linoleic acid doses were used as an explanatory variable and colonies were treated as a random effect. Also, we compared the proportions of the live or dead non-soldier aphids pushed by soldier(s) among treatments, using GLMM with sequential Bonferroni (*P* < 0.05). The proportion of pushed aphids was regarded as the response variable, assuming a binomial distribution. Treatments were used as an explanatory variable and colonies and replicates were treated as random effects.

## Results and discussion

### Soldiers push dead aphids selectively

**Fig. 1:**
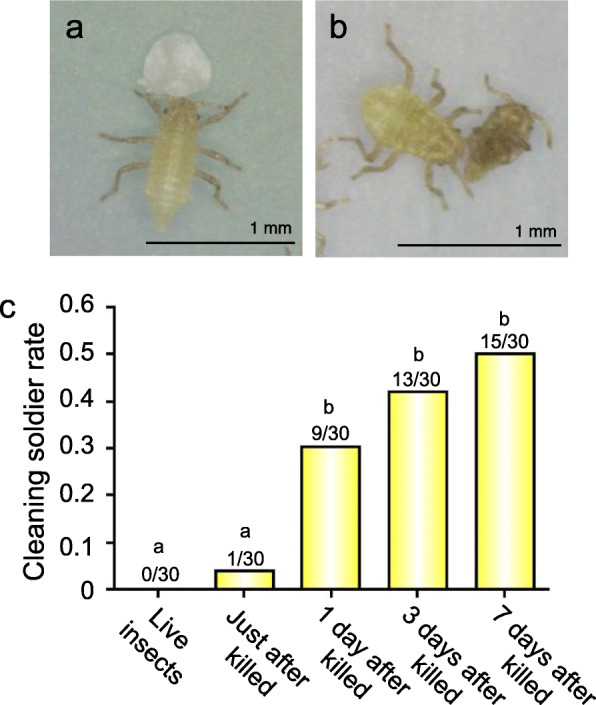
Cleaning behavior of soldiers of *T. styraci*. (**a**) A soldier pushing a honeydew globule. Also see Movie 1. (**b**) A soldier pushing a dead aphid. Also see Movie 2. (**c**) Proportion of soldiers that exhibited the cleaning behavior against live old adults or dead old adults that were 0, 1, 3 and 7 days after freeze-killing. Different alphabetical letters (a–b) indicate statistically significant differences (GLMM with sequential Bonferroni; *P* < 0.05). In total, 150 young soldiers from 3 galls were subjected to the behavioral observations

As previously reported [26], soldiers of *T. styraci* exhibited the typical cleaning behavior, pushing colony wastes such as honeydew globules, shed skins and aphid cadavers with their head continuously, even on the artificial diet plates (Fig. 1a and b; Movies 3 and 4). Here it should be noted that live aphids were not pushed but dead aphids were actively pushed by soldiers (Fig. 1c). While freshly killed aphids elicited the cleaning behavior only weakly, a day after killing and on, the dead aphids strongly elicited the soldier’s cleaning behavior (Fig. 1c).

### Linoleic acid seeps out on body surface of dead aphids

**Fig. 2:**
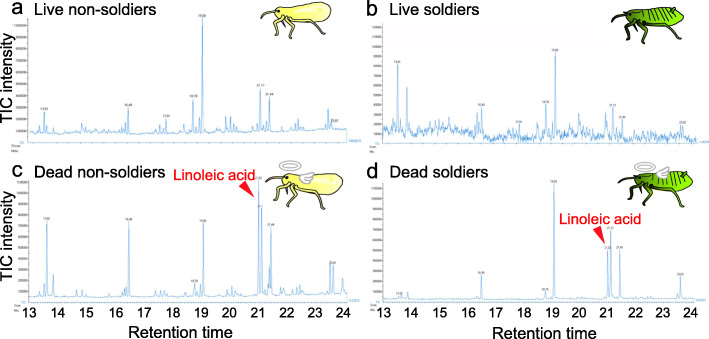
Fatty acid profiles on the body surface of *T. styraci*. (**a**) Live old adults. (**b**) Live old soldiers. (**c**) Dead old adults 3 days or longer after freeze-killing. (**d**). Dead old soldiers 3 to 7 days after freeze-killing. Retention times are expressed in minutes. TIC: total ion current

**Fig. 3:**
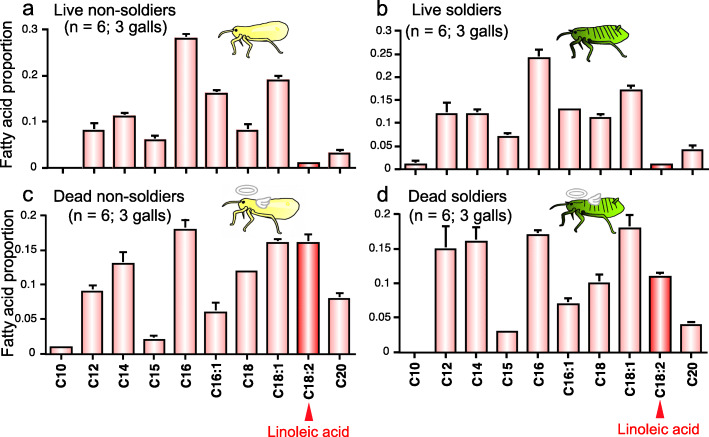
Relative quantities of fatty acids on the body surface of *T. styraci*. (**a**) Live old adults. (**b**) Live old soldiers. (**c**) Dead old adults 3 to 7 days after freeze-killing. (**d**). Dead old soldiers 3 days or longer after freeze-killing. Statistically significant differences were detected in linoleic acid proportion between the live and dead adults (Wilcoxon rank-sum test; *P* < 0.05) and between the live and dead soldiers (Wilcoxon rank-sum test; *P* < 0.005). Sample sizes are shown in parentheses and standard errors are shown by vertical bars

**Fig. 4:**
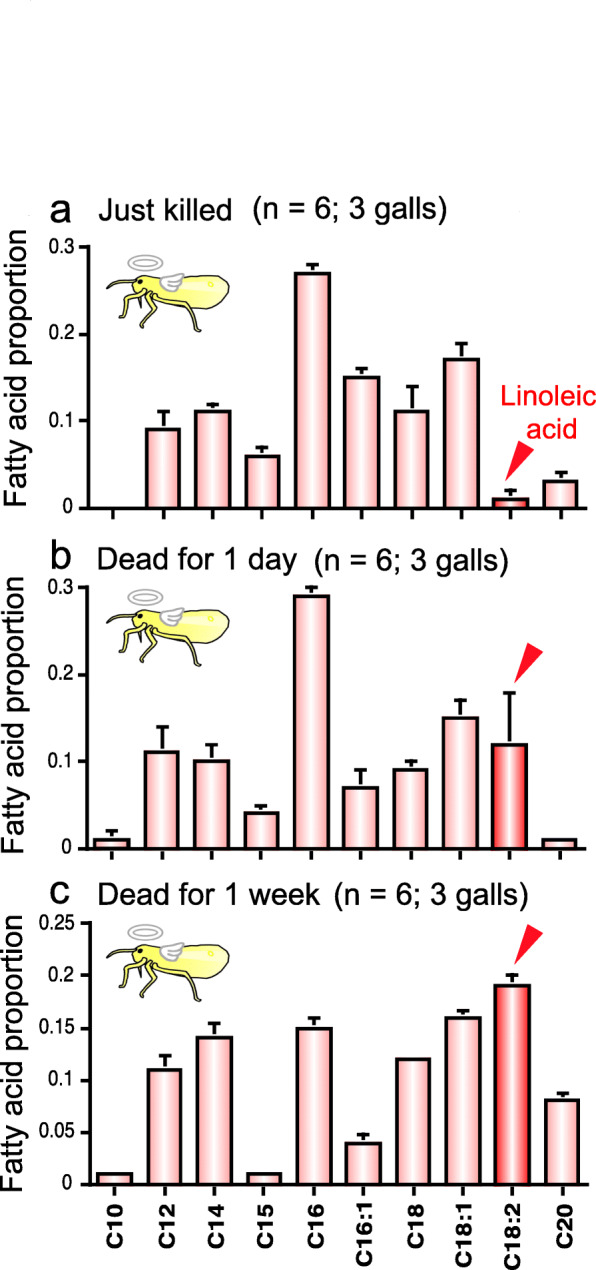
Relative quantities of fatty acids on the body surface of dead old adults of *T. styraci*. (**a**) 0 day, (**b**) 1 day, and (**c**) 1 week after freeze-killing. The relative quantity of linoleic acid increased significantly after death, resulting in tenfold increase for the first one day (Steel’s multiple comparison test; *P* = 0.06) and twentyfold increase for one week after death (Steel’s multiple comparison test; *P* < 0.05). Sample sizes are shown in parentheses and standard errors are shown by vertical bars

What differences between the live and dead aphids are relevant to the different behavioral responses of soldiers? Considering that fatty acids are known to function as corpse recognition factors in other social insects such as ants, bees and termites [27–34], we extracted the body surface of live soldiers and non-soldiers as well as dead soldiers and non-soldiers with hexane, and analyzed the extracted fatty acid samples by GC-MS and GC-FID. Regardless of soldiers or non-soldiers, the dead aphids exhibited a significant peak of linoleic acid, which was scarcely detected in the live aphids (Figs. 2 and 3). While little linoleic acid was found on the body surface of freshly killed aphids, significant quantities of linoleic acid were detected on the body surface of the dead aphids one day or one week after killing (Fig. 4). These results indicated that linoleic acid seeps out on the body surface after the aphids die.

### Linoleic acid triggers soldier’s cleaning behavior

**Fig. 5:**
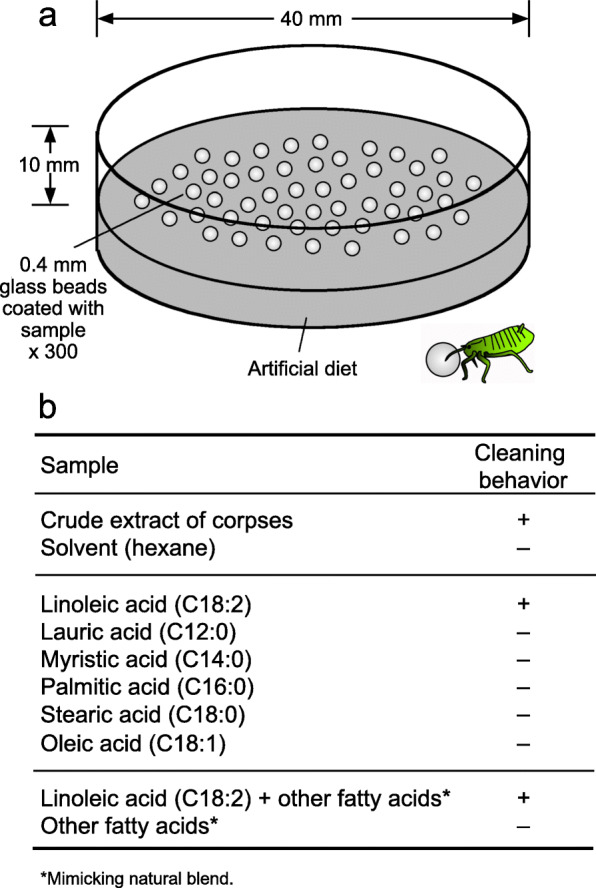
Bioassay of factors that trigger the cleaning behavior of soldiers of *T. styraci*. (**a**) Schematic illustration of the experimental system. (**b**) Results of the bioassay (also see Fig. 6). Lauric acid, myristic acid, palmitic acid, stearic acid, oleic acid and linoleic acid were tested alone or blended at the ratios of 0.6: 0.8: 1.1: 0.8: 1.0: 1.0, which mimick the cuticular fatty acid profile of 1-week-old dead adult aphids

Is linoleic acid on the body surface of the dead aphids relevant to the soldier’s cleaning behavior? To verify this idea, we performed soldier’s behavioral assay using sample-coated glass beads. On an artificial diet arena, soldiers were placed with 0.4 mm glass beads, which had been soaked in a hexane-extracted sample and then dried, and their behaviors against the glass beads were observed (Fig. 5a). Crude extract of dead aphids, pure linoleic acid, and fatty acid mixtures containing linoleic acid elicited the soldier’s cleaning behavior, whereas the other fatty acids and their mixtures did not (Fig. 5b), which confirmed that linoleic acid is certainly the molecular factor that triggers soldier’s cleaning behavior.

### Levels of cleaning behavior dependent on aphid castes and linoleic acid doses

**Fig. 6:**
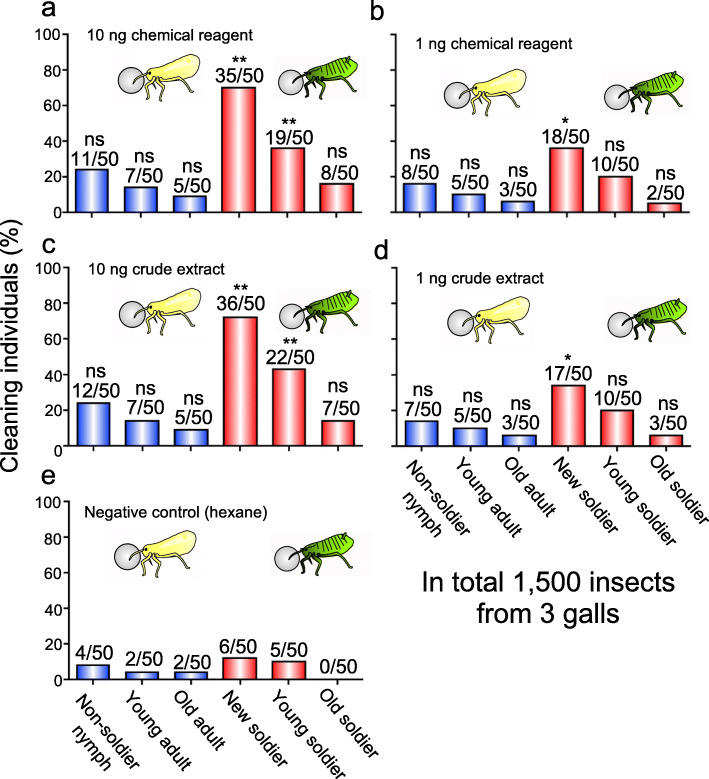
Proportion of cleaning non-soldiers and soldiers of *T. styraci* against sample-coated glass beads. (**a**) Glass beads treated with 3000 ng linoleic acid per 20 μl hexane. The test sample applied to individual glass beads is equivalent to 10 ng linoleic acid per bead. (**b**) Glass beads treated with 300 ng linoleic acid per 20 μl hexane. The test sample applied to individual glass beads is equivalent to 1 ng linoleic acid per bead. (**c**) Glass beads treated with 1 x crude surface extract (500 dead old adults per 20 μl hexane). The test sample applied to individual glass beads is equivalent to about 10 ng linoleic acid per bead. (**d**) Glass beads treated with 0.1 x crude surface extract (50 dead old adults per 20 μl hexane). The test sample applied to individual glass beads is equivalent to about 1 ng linoleic acid per bead. Asterisks indicate statistically significant differences as compared with the counterpart control group (GLMM with sequential Bonferroni: *, *P* < 0.05; **, *P* < 0.01; ns, not significant). In total, 1500 insects from 3 galls, which contained non-soldier nymphs, unwinged adults, and second instar soldiers, were subjected to behavioral observations: non-soldier nymphs, second or third instar nymphs; young adults, adults within 7 days after final molt; old adults, adults of 8 days or older after final molt; new soldiers, soldiers within one day after molt; young soldiers, soldiers 10 days after molt; old soldiers, soldiers 20 days after second instar molt

Our previous study showed that, in *T. styraci*, not only soldiers but also non-soldiers perform the cleaning behavior, though less frequently, and that young soldiers perform the cleaning behavior more frequently than old soldiers [26]. These patterns were generally observed when glass beads coated with linoleic acid or aphid surface extract were presented to the insects on the artificial diet arena: (i) soldiers exhibited the cleaning behavior more frequently than non-soldiers; (ii) young soldiers performed the cleaning behavior more frequently than old soldiers; and (iii) the higher the concentration of linoleic acid was, the more active cleaning behavior was induced (Fig. 6). These observations reinforced the idea that linoleic acid is the molecular factor that triggers the cleaning behavior in the social aphid *T. styraci*.

### Soldier’s cleaning behavior against live and dead aphids treated with linoleic acid

**Fig. 7:**
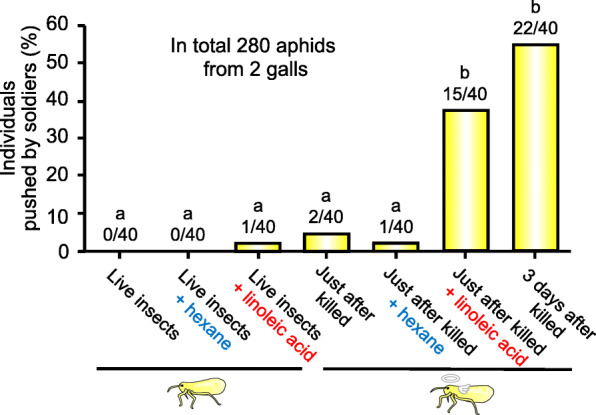
Cleaning behavior of soldiers of *T. styraci* against live and dead aphids treated with linoleic acid. For each experimental treatment, 10 non-soldier test insects were introduced into the artificial diet arena containing 100 young soldiers, and continuously observed for 30 min to monitor whether they are pushed by soldiers or not. Live non-soldier aphids and freshly killed non-soldier aphids were subjected to no treatment as negative control, hexane treatment as solvent control, and linoleic acid treatment, which were replicated 4 times respectively. Dead non-soldier aphids 3 days after killing were also examined as positive control. In total, 280 non-soldier test insects derived from two galls were subjected to the experiments. The numbers of non-soldier insects pushed by soldier(s) were compared between the experimental treatments. Different alphabetical letters (**a–b**) indicate statistically significant differences (GLMM with sequential Bonferroni; *P* < 0.05)

When treated with linoleic acid, freshly killed aphids were actively pushed by young soldiers, whereas live aphids seldom elicited the soldier’s cleaning behavior (Fig. 7). These results indicated that linoleic acid certainly acts as a corpse recognition factor when smeared on immobile dead aphids, but the action is cancelled when smeared on mobile live aphids. Considering that freshly killed aphids elicit the soldier’s cleaning behavior when treated with linoleic acid, it seems plausible that movement of the live aphids suppresses the soldier’s cleaning behavior even in the presence of linoleic acid.

### Origin of linoleic acid in dead aphids

**Fig. 8:**
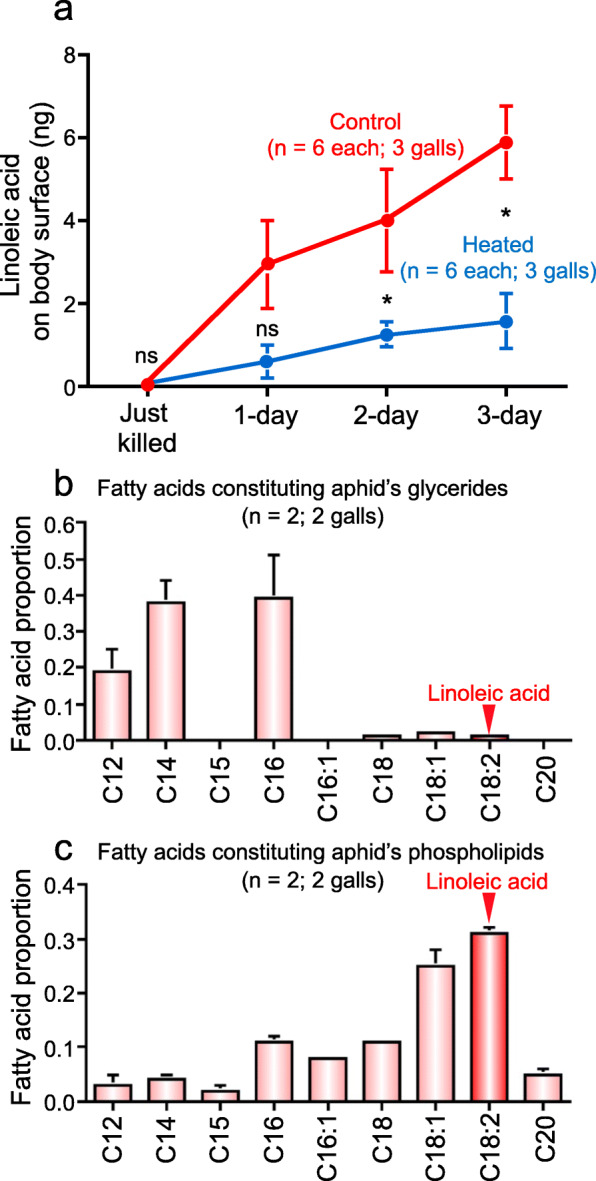
Origin of linoleic acid on the body surface of *T. styraci*. (**a**) Relative quantities of linoleic acid on the body surface of dead old adults with or without heating by microwave after freeze-killing. Asterisks indicate statistically significant differences (*P* < 0.05) as compared with the control group (Wilcoxon rank-sum tests: *, *P* < 0.05; ns, not significant). (**b**) Relative quantities of fatty acids released by hydrolysis of glycerides extracted from live old adults*.* (**c**) Relative quantities of fatty acids released by hydrolysis of phospholipids extracted from live old adults*.* Sample sizes are shown in parentheses and standard errors are shown by vertical bars

Where does linoleic acid on the body surface of dead aphids come from? By heating the dead aphids by microwave, levels of linoleic acid on the body surface were significantly suppressed in comparison with the control dead aphids (Fig. 8a), which suggested the possibility that linoleic acid may be produced via autolytic enzymatic reactions in the dead aphids. We extracted glycerides and phospholipids from *T. styraci*, hydrolyzed them, and analyzed the resultant fatty acids by GC-MS and GC-FID, which revealed that linoleic acid was more abundant in phospholipids than in glycerides (Fig. 8b and c). In insect cells and tissues, the majority of glycerides are stored in fat body cells as cytoplasmic lipid droplets, whereas most phospholipids are present as the major constituent of cell membranes [35]. On the basis of these results, we suggest that, in dead aphids, linoleic acid is produced by enzymatic autolysis of cell membrane phospholipids, seeps out on the body surface, and functions as a chemical signal of dead colony mates.

## Conclusions

In conclusion, we identified the specific fatty acid, linoleic acid, as corpse recognition signal of the social aphid *T. styraci*. Linoleic acid is not present on the body surface of live aphids, generated by enzymatic autolysis of cell membranes in dead aphids, and seeping out on the body surface of aphid cadavers, thereby triggering the cleaning behavior of soldiers. This study presents the first report of death pheromone from social aphids. It is of evolutionary interest why phylogenetically unrelated social insects, ants, bees (Hymenoptera), termites (Blattodea) and aphids (Hemiptera), have adopted the common unsaturated fatty acids, oleic acid and/or linoleic acid, as chemical cues for detecting dead colony mates [27–34]. In this context, it is notable that oleic acid and/or linoleic acid are also reported to elicit avoidance responses in non-social insects and allied arthropods like cockroaches, caterpillars, collembolans, woodlice and pillbugs [36, 37]. Plausibly, these unsaturated fatty acids are generally produced via enzymatic autolysis of cell membranes in dead arthropods and therefore amenable to utilization as a reliable signal of death, which highlights an impressive example of evolutionary parallelism underpinning diverse ecological and behavioral interactions among them. Considering that soldiers and their cleaning behaviors have repeatedly evolved in aphids [7–9], whether linoleic acid is also used as corpse recognition signal in other social aphid lineages is of evolutionary interest. Finally, we note that the results obtained in this study on the artificial diet rearing system should be verified in natural galls in future studies.

## Supplementary Information

**Additional file 1: Movie 1.** Soldiers of gall-forming social aphid *Tuberaphis styraci* disposing honeydew balls from an opening of a natural gall.

**Additional file 2: Movie 2.** Soldiers of gall-forming social aphid *Tuberaphis styraci* disposing an aphid cadaver from an opening of a natural gall.

**Additional file 3: Movie 3.** Soldiers of gall-forming social aphid *Tuberaphis styraci* cleaning honeydew balls on an artificial diet plate.

**Additional file 4: Movie 4.** Soldiers of gall-forming social aphid *Tuberaphis styraci* cleaning aphid cadavers on an artificial diet plate.

## Data Availability

Not applicable.

## Abbreviations

GC-MS: Gas chromatography-mass spectrometry
GC-FID: Gas chromatography with a flame ionization detector
